# Building a Casimir metrology platform with a commercial MEMS sensor

**DOI:** 10.1038/s41378-019-0054-5

**Published:** 2019-04-22

**Authors:** Alexander Stange, Matthias Imboden, Josh Javor, Lawrence K. Barrett, David J. Bishop

**Affiliations:** 1 Division of Material Science and Engineering, Boston University, Boston, MA 02215 USA; 2 Institute of Microengineering, École Polytechnique Fédérale de Lausanne, Neuchâtel, 2000 Switzerland; 3 Department of Mechanical Engineering, Boston University, Boston, MA 02215 USA; 4 Department of Physics, Boston University, Boston, MA 02215 USA; 5 Department of Electrical and Computer Engineering, Boston University, Boston, MA 02215 USA; 6 Department of Biomedical Engineering, Boston University, Boston, MA 02215 USA

**Keywords:** Sensors, Electrical and electronic engineering

## Abstract

The Casimir Effect is a physical manifestation of quantum fluctuations of the electromagnetic vacuum. When two metal plates are placed close together, typically much less than a micron, the long wavelength modes between them are frozen out, giving rise to a net attractive force between the plates, scaling as *d*^−4^ (or *d*^−3^ for a spherical-planar geometry) even when they are not electrically charged. In this paper, we observe the Casimir Effect in ambient conditions using a modified capacitive micro-electromechanical system (MEMS) sensor. Using a feedback-assisted pick-and-place assembly process, we are able to attach various microstructures onto the post-release MEMS, converting it from an inertial force sensor to a direct force measurement platform with pN (piconewton) resolution. With this system we are able to directly measure the Casimir force between a silver-coated microsphere and gold-coated silicon plate. This device is a step towards leveraging the Casimir Effect for cheap, sensitive, room temperature quantum metrology.

## Micro-electromechanical Systems: Applying the Casimir effect in a commercial sensor

The Casimir effect is a quantum fluctuation force that exists between conducting surfaces separated by hundreds of nanometers, and the effect holds promise for application as a sensing tool in conjunction with an inexpensive micro-electromechanical system (MEMS) device. A team headed by Alexander Stange in the Bishop group at Boston University, United States, observed the Casimir effect under ambient conditions using a modified off-the-shelf MEMS sensor. The authors were able to integrate a Casimir cavity with the MEMS device by attaching various microstructures onto the MEMS sensor, converting it into a customized force measurement platform. The team believes that the Casimir effect has considerable potential as a practical, controllable sensing tool and that it could be used for such purposes as temperature sensing, AC voltage measurements, and low-impedance current measurements.

## Introduction

One of the most commonly used technologies enabled by micro-electromechanical systems (MEMS) are inertial sensors^1,2^. These devices are a part of our everyday lives, from sensing the orientation of smartphones (low *g*) to detecting collisions in automobiles in order to deploy airbags (high *g*). Current state-of-the-art low *g* MEMS inertial sensors are capable of sensing sub milli*-g* accelerations with noise densities of around 0.1 mg/Hz^1/2^ or less^3–5^. The proof-mass of these sensors typically weigh around 1 μg, meaning the devices are capable of resolving forces below 1 pN. Such force sensitivity is comparable to the performance of an atomic force microscope (AFM), but is realized on a single millimeter-scale chip and costs just tens of dollars per device. In this work, we show that by attaching a silver-coated microsphere to its proof-mass, a commercial MEMS inertial sensor can measure the Casimir force that is exerted onto the microsphere, and therefore directly onto the proof-mass of the sensor, due to its interaction with an external metallized plate.

The Casimir Effect, first derived by Hendrik Casimir in 1948, is a quantum fluctuation force that exists between conducting surfaces separated by hundreds of nanometers^6^. In its simplest case (two perfectly smooth, perfectly conducting planar surfaces), the Casimir Effect manifests itself as an attractive force between the two objects, which scales as one over separation to the fourth power. The physical origin of this phenomenon is purely quantum mechanical, arising from zero-point fluctuations exerting a net pressure on the conducting surfaces. Because of the small scale of this effect (pN forces at nanometer separations), Casimir force detection nearly always involves a micromechanical system of some kind. Most commonly a modified AFM setup is used, in which a cantilever is adapted to measure forces exerted on it due to Casimir interactions^7–10^. Other work using MEMS has made use of torsional resonators^11–13^. Additionally, devices that can integrate both Casimir surfaces onto a single chip^14^ are less prone to low-frequency noise and thermal drift due to smaller components and higher mechanical resonant modes, but come at the cost of reduced interaction area and limited separation ranges.

The main advantage of a modified commercial MEMS sensor is the pre-optimized design of the MEMS, and the supporting integrated circuitry simplify the device fabrication and apparatus immensely. Despite using an external piezo-mounted plate, we are able to clearly see the Casimir force in ambient conditions. Compared to AFM, the size and cost is superior by orders of magnitude and the linear transduction of an applied force to an electronic output signal is a built-in feature of the sensor.

The ability to measure the Casimir Effect with commercial MEMS devices is an exciting prospect because it indicates that this effect could be used as a practical, controllable engineering tool within a MEMS system. For example, it has been suggested that a MEMS oscillator parametrically driven by the Casimir force would exhibit a gain that scales as one over the Casimir cavity size to the fifth power or as applied direct current (DC) voltage to the tenth power^15^. In addition to providing a means of investigating the Casimir Effect itself, this system could be useful for temperature sensing, alternate current (AC) voltage measurements, low-impedance current measurements, or probing any measurand, which could be coupled into a physical movement of the sensor proof-mass. Successfully integrating a Casimir cavity into a well-developed, scalable MEMS device is an important step in realizing Casimir-enabled sensors as a practical, room temperature quantum metrology tool.

## Results

### Casimir cavity integration with MEMS

#### Micro-gluing onto post-release MEMS

**Fig. 1:**
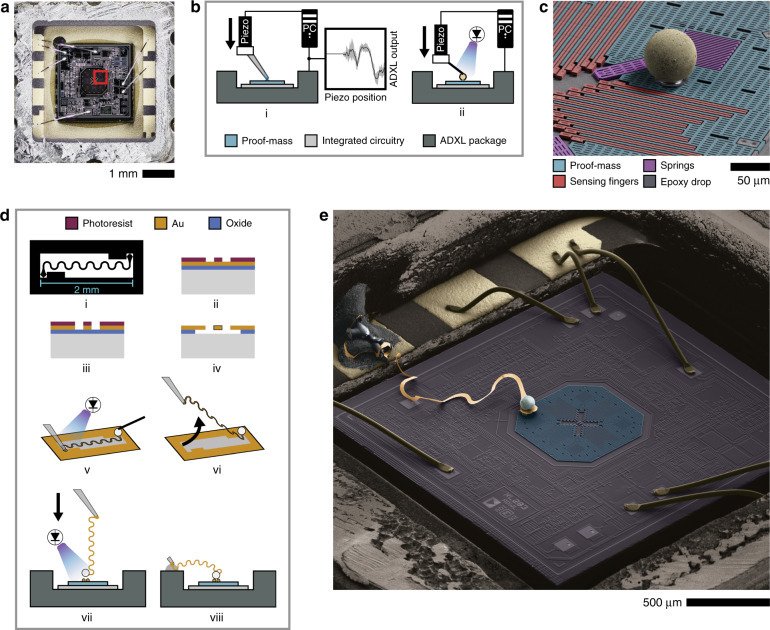
Modification of post-release MEMS sensor. **a** Top-view optical image of the ADXL203 die inside the package with the lid removed. The octagonal proof-mass can be seen in the center. Highlighted red box indicates the area of the proof-mass shown in the scanning electron microscope (SEM) image in (**c**). **b** Schematic of feedback-assisted attachment of microspheres onto the proof-mass. (i) ADXL output is monitored while a piezoelectric actuator lowers a micro-pipette (30 µm tip diameter) containing epoxy. Upon contact, surface forces draw out a few picoliters of epoxy and the pipette is automatically retracted. (ii) Schematic of sphere placement. ADXL output monitored as before while the sphere is lowered into the droplet. Once contact is made, the epoxy is cured by ultraviolet (UV) exposure. **c** Colorized SEM image of one quadrant of the micro-electromechanical system (MEMS) with a microsphere glued to the proof-mass using the micro-gluing technique. The interdigitated sensing electrodes and anchoring springs of the proof-mass can also be seen. All of the MEMS structures are 4 µm thick. **d** Schematic of device assembly steps. The lithography mask (i) for the nano-ribbon wire is designed with a 2 mm nominal length, 25 µm lateral width, and a 58 µm radius circle at each end for attachment. After fabricating the wires out of a 500 nm layer of evaporated Au on oxide with standard lithography and etching (ii–iv), the device is assembled (v–viii) by attaching a microsphere to one end of the wire, peeling the wire off the substrate with a pipette, then lowering the Au nano-ribbon wire (with the Ag sphere attached) onto two smaller support spheres, which have been previously bonded to the ADXL203 proof-mass using the micro-gluing technique shown in (**b**). **e** Colorized SEM image of an example of a fully modified ADXL203 (not the device used in this work)

Because the modification involves bonding objects to a post-release MEMS device, great care must be taken in keeping mechanical forces exerted on the freely moving parts to a minimum. We present a technique that allows us to glue microspheres directly to the proof-mass of a MEMS inertial sensor without compromising its functionality. In this work, we use an accelerometer from Analog Devices (ADXL203), pictured in Fig. 1a.

Outlined in Fig. 1b is our process which involves depositing picoliter volume droplets of ultraviolet (UV) curable epoxy using a micro-pipette attached to a piezoelectric actuator onto the proof-mass then placing a microsphere onto the droplet using a probe tip (contact forces are sufficient to pick up the microsphere). The pipette or probe tip can be moved in plane with a micromanipulator, while the Z position is controlled with nanometer precision using the piezoelectric actuator. The advantage of assembling onto post-release MEMS is that we can sense when contact with the proof-mass occurs by actively monitoring the noise on the outputs of the device (see Fig. 1b). This feedback is what allows us to deposit droplets gently onto the proof-mass without forcing liquid into the release holes or breaking the springs. As can be seen in Fig. 1c, the proof-mass (shown in blue) provides only a few small areas over which droplets can be placed without interfering with other parts of the MEMS such as the sensing fingers or the springs. In order to attach larger objects, we use one or more spheres (Au-coated solid barium titanate glass) as supports for other objects to be set upon, like legs of a table. Once these “legs” are formed, one can then attach a wide variety of microscale objects, provided they do not interfere with the MEMS and are able to be picked up and placed gently. For example, it is possible to place a sub-millimeter neodymium rare-earth magnet on top of the support spheres for high-resolution gradient magnetometry (on going work). For the device presented in this paper, two 30 µm diameter solid spheres were glued onto the proof-mass as a platform for the rest of the assembly discussed below.

#### Casimir force detection

The functional component of our device is a conductive microsphere attached to the proof-mass of the inertial sensor that forms one-half of the Casimir cavity. Doing this results in a device that can not only measure accelerations applied to the device body (as was originally intended) but also forces exerted directly onto the sphere, namely electrostatic and Casimir forces. The sphere is 55 µm in radius and made of hollow borosilicate glass coated with 50 nm of Ag and has a mass of roughly 0.1 µg. It was found that Ag-coated hollow spheres had much lower surface roughness than Au-coated solid spheres (Supplementary information S1), so an Ag sphere was used in the Casimir cavity, while two smaller Au spheres (about 0.5 µg each) were used as supports. It should be noted that the mass added to the proof-mass does not affect the functionality of the device at DC. For dynamic measurements, however, the added mass does lower the overall bandwidth of the device.

One requirement in any Casimir device is the ability to control the electric potential on the interacting surfaces. This is due to the presence of residual electrostatic forces, which are caused by trapped charges, adsorbates, and the poly-crystalline nature of the metallic surfaces^16–18^. The latter results in local differences in the work function of the materials (also known as patch potentials), which sum up to a non-zero effective potential difference, even when the materials are electrically connected^19^. This overall residual potential is a common source of error in Casimir force measurements if not controlled for. To do this, a 500-nm-thick serpentine ribbon wire connects the surface of the Ag microsphere to an open bonding pad on the ceramic package. The conductivity of the wire provides a means of controlling the voltage on the sphere and its flexible geometry ensures a low spring constant, thus allowing for minimal restriction of the motion of the proof-mass and the restoring force of the polysilicon springs. The effect that the wire has on the overall effective spring constant of the system (and therefore the force sensitivity) is minimal and easily accounted for as the post-modification force sensitivity is re-calibrated by using known electrostatic forces between the sphere and the plate. Additionally, because the electrostatic calibration is done with the sphere and plate in the same configuration as the Casimir force measurement, any rotational movement of the proof-mass due to torque applied from the sphere (which is not perfectly centered) will be accounted for. The process of assembling this device is shown schematically in Fig. 1d and discussed further in Methods.

**Fig. 2:**
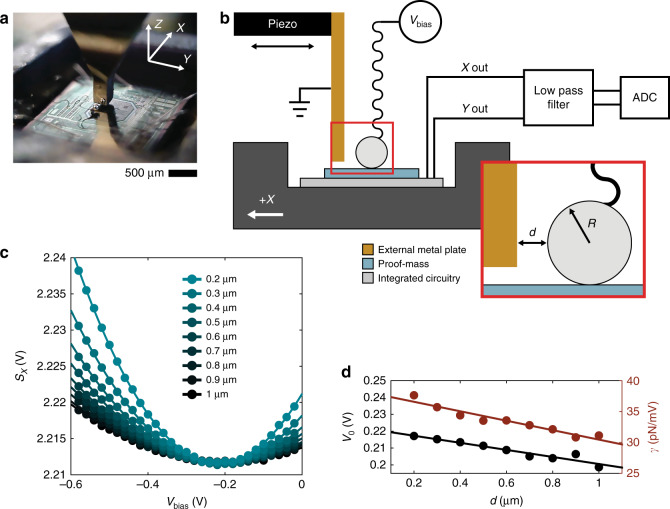
Apparatus and calibration. **a** Optical image of the modified ADXL203 used to collect the data presented in this paper. Also pictured is the external Au-coated plate mounted on a piezoelectric actuator (out of frame). **b** Schematic of full setup. The sensor X and Y outputs are fed through an 8-pole low-pass filter with a 3 Hz cutoff to isolate the desired direct current (DC) signal and then read by a 16-bit Analog to Digital Converter (ADC). The Casimir force acts along the X direction for this particular device. INSET: Diagram of Casimir cavity geometry showing sphere-plate separation (*d*) and sphere radius (*R*). For simplicity, the two support spheres are not pictured. In reality, the Ag sphere is sitting 20–30 µm above the proof-mass. **c** Sensor signal data as *V*_bias_ is varied at different separations. Circles are measured data and the solid lines are second order polynomial fits to the data. **d**. *V*_0_ and *γ* versus separation. These values are computed from the minima and curvature of parabolas fit to data in Fig. 2c

The second half of the Casimir cavity is formed from a Au-coated plate mounted on a linear piezoelectric actuator. For the device used in this paper, the plate approaches the sphere along the *X*-axis of the sensor. The entire setup is mounted on an optical breadboard and contained in a temperature-controlled enclosure on top of an active vibration isolation table. A schematic of the device and apparatus can be seen in Fig. 2b.

### Device performance

#### Electrostatic characterization of residual potential and force calibration

Electrostatic forces are used to measure the residual potential difference, *V*_0_, between the sphere and the plate and to calibrate the force sensitivity, *γ*, which relates the sensor output voltage, *S*, to the applied force according to *F* = *γS*. In Fig. 2c we plot the voltage output in the X direction, *S*_X_, as we vary the potential applied between the grounded plate and the microsphere (*V*_bias_) at different separations between 200 nm and 1 µm where electrostatic forces are much larger than the Casimir force. The minima of these voltage sweeps indicate the bias that cancels residual potential between the metal surfaces, and the curvature of the sweeps give a calibration coefficient between the sensor voltage output and force, which can then be used for Casimir force measurements (see further discussion on this approach in Methods).

Both the residual potential and the force sensitivity of the device appear to be functions of separation and are approximately linear. Over the full 800 nm scan range, it is observed that *V*_0_ varies by 9% and *γ* varies by 20%, with average values of 0.21 V and 33.5 pN/mV, respectively.

### Casimir force measurement

**Fig. 3:**
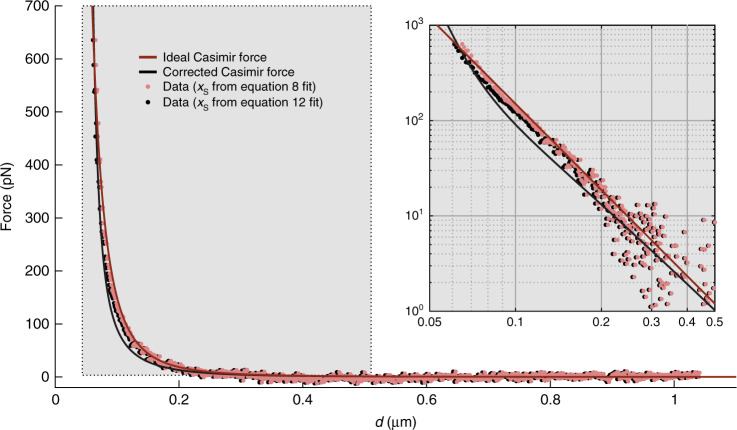
Casimir force measurements compared with ideal theory (Eq. 8) (red) and theory for real metals (Eq. 12) (black). Inset shows the highlighted section of the data in log–log scale for better comparison between data and theory at small separations. The two sets of data are identical but shifted by 2 nm along the abscissa because of the different values of *x*_s_ returned from the fits to Eqs. 8 and 12

The data in Fig. 3 is a measurement of the force applied to the proof-mass along its *X* axis as a function of separation between the Ag-coated microsphere (which is attached directly to the proof-mass) and an external Au-coated plate. Electrostatic contributions have been minimized according to the methodology discussed in the following sections. The red and black data points are the same set of data fit to either the ideal Casimir force theory (solid red line) given by Eq. 8 or the corrected Casimir force theory (solid black line) given by Eq. 12 with only *x*_s_ (i.e., where separation *d* = 0) as a free parameter. According to the ideal fit, the last measured data point at 635.5pN is 65 nm away from *d* = 0. According to the corrected Casimir fit, the last measured data point is 63 nm away from *d* = 0.

## Discussion

### Distance dependence of residual potential and sensitivity

The separation dependence of the residual potential is a well-known occurrence^20–22^ as the potential measured is an effective sum of the contributions from different regions across the surfaces of each metal. As the separation changes, these contributions will sum differently due to the inverse square dependence of the electrostatic force. The linear dependence of the force sensitivity of the device is due to the interaction of the grounded Si plate with the fringe fields of the interdigitated capacitor fingers of the sensor. The capacitive sensing relies on a small AC signal applied between the fixed fingers and the movable fingers. Because the plate is held at the same ground as the device, it will deflect fringe field lines from this applied voltage and result in an out-of-plane force exerted on the grounded fingers^23,24^. As the plate’s position is varied it will overlap with more fringe fields, thus exerting a larger out of plane force and decreasing the sensitivity. This effect is more prominent the closer the plate is to the fingers (see Supplementary information S2). Therefore, there is an ideal range of plate heights at which the experiment can be performed, where the fringe field interactions are minimized while also ensuring adequate area of interaction between the side of the sphere and the plate.

### Casimir force comparison with theory

In Fig. 3 it can be observed that the measured data is modeled more accurately by the ideal Casimir theory (root mean squared error of 7.4pN) compared to the corrected theory (root mean squared error of 10.5pN). While the corrected theory takes into account the finite conductivity and non-zero temperature of the surfaces, the perturbation approach used in ref. ^25^ is in fact only valid for separations ranging from a few hundred nanometers to several micrometers. At short separations, the dielectric permittivity of the respective metals at high frequencies contributes to the force magnitude and changes the scaling with separation (V.M. Mostepanenko, personal communication, October 26, 2018). As a result, although the ideal theory overestimates the measured force in the 80 to 200 nm range, the corrected model in Eq. 12 underestimates it by far greater. More accurate fitting may be possible by performing a numerical calculation of the Lifshitz formula using optical data for the complex index of refraction of the metal surfaces, but such analysis is outside the scope of this work.

Considering random measurement errors, the linear fits shown in Fig. 2d returned root mean square errors to the data of 1.8 mV and 0.6pN/mV for *V*_0_ and *γ*, respectively. Assuming a mis-calculated *V*_0_ off by three standard deviations (5.4 mV) at the closest point of approach of 60 nm, we would be introducing an unwanted additional electrostatic force of 0.74 pN, which is just 0.1% of the Casimir force measured at that point. This value is also much less than the random error in the force measurement due to uncertainty in the product *γS*_X_ (whose combined errors propagate to as high as 60 pN at the closest measured point). It is therefore likely that the discrepancy between measurement and theory in this distance range are not due to imperfectly canceled electrostatic forces, as the Casimir force is the dominant interaction.

The reason for this discrepancy is most likely due to the assumptions of the geometry of the cavity, both at the microscale (i.e., sphere and plate shapes and arrangement) and at the nanoscale (surface roughness). The sphere radius, *R*, is used in both the electrostatic calibration analysis as well as the theoretical Casimir fits. In both cases, we use *R* in the framework of the proximity force approximation (PFA), which assumes a perfectly spherical surface and an infinite plane in the *d* « *R* limit^26^. Because of this, both the calibration factor and the Casimir force are proportional to *R*, so any errors in the value of *R* (which was measured optically) do not affect the fitting. However, if the sphere is not perfectly spherical, then we would expect both the electrostatic force and the Casimir force to scale differently depending on the exact shape^27^. Additionally, due to physical constraints of our setup, the plate is limited to extend only ∼80–90 µm below the central plane of the sphere. Because the PFA assumes an infinite plane, this asymmetry may result in a systematically overestimated force sensitivity of the device.

The surface roughness also becomes very important in Casimir interactions at separations <∼ 100 nm^28,29^. The AFM scans taken were on separate samples (see supplementary information S1), which went through the same coating processes as the sphere and plate used in this device. While this may be useful for capturing average roughness values, it is not specific to this exact cavity, which may have extreme asperities that cause deviation from the expected scaling below 100 nm separations. To improve both of these geometry-related systematic uncertainties, characterization of individual spheres would need to be done prior to subsequent assembly.

Nevertheless, our results show that an Ag surface and an Au surface in our atmospheric MEMS system exhibit an interaction that can be described quite well by the ideal Casimir force model. This is a promising finding as we move forward with Casimir-enabled sensing devices, such as that described by Imboden *et al*^15^. The results presented here imply that approximating this interaction as a simple inverse cubic relation is quite sufficient for further analysis and modeling.

## Conclusions

The purpose of this work was two-fold: First, we have successfully integrated a Casimir cavity onto a MEMS system, which is capable of resolving piconewton forces. This system provides the experimenter with a customizable apparatus for investigating the Casimir Effect with non-trivial geometries, materials, or surface morphologies such as nanostructures or chiral metamaterials^30–34^. Additionally, this work is an important stepping stone in our goal to leverage the extraordinary distance dependence of the Casimir force to provide an enhancement in the sensitivity of the device through parametric techniques. Second, we have shown that it is possible to perform highly sensitive quantum metrology in ambient conditions with off-the-shelf consumer MEMS sensors, which are widely available and very inexpensive. Once functionalized, these devices can be used as a novel tool for experimenters—a literal platform capable of performing a variety of interesting micro- and nanoscale low-force experiments. Using the feedback-assisted micro-gluing process we have developed, one can re-purpose the sensor to transduce any measurand that can be coupled into a displacement of the proof-mass.

## Materials and methods

### MEMS sensor

The fundamental building block of our Casimir measurement system is a MEMS accelerometer from Analog Devices (ADXL203)—a two-axis capacitive accelerometer with analog voltage outputs for the in-plane X and Y directions with a sensitivity of ∼ 1 V/*g* along both axes^5^. The polysilicon MEMS consists of an octagonal proof-mass (760 µm in width and 4 µm in thickness) that is anchored to the substrate via four serpentine springs with a total effective spring constant of roughly 1 N/m. The proof-mass/spring system has a fundamental resonant mode at 5.4 kHz with a quality factor of 10 in air and 1000 in vacuum (∼1 × 10^−4^ Torr). The proof-mass also has four sets of finger electrodes, forming an interdigitated differential capacitor with another set of fingers anchored to the substrate. Any force applied to the proof-mass moves it, thus changing this capacitance. The proof-mass is surrounded by integrated circuitry on the same chip that demodulates the signals from the differential capacitance measurements and rectifies them into two independent output voltages (nominally 2.5 V with zero applied acceleration) that are proportional to the position of the proof-mass in the X and Y directions. Because the springs obey Hooke’s Law, the outputs are linearly proportional to the forces on the proof-mass. This platform has been optimized with its sensing circuit integrated with the MEMS process to produce a very low noise system that can resolve forces of ∼1pN applied directly to the proof-mass. The package is easily opened with a straight edge razor blade. After the lid is removed, the MEMS proof-mass and electronics can be seen as shown in Fig. 1a.

### Device assembly

To assemble this device, the following process (outlined in Fig. 1d) was developed: (1) a serpentine wire with circular ends is fabricated by lithographically patterning and etching a 500-nm-thick layer of Au on top of a 2 µm layer of thermally grown oxide. The wire is then released by removing the oxide in hydrofluoric acid (i–iv). 2) A single 55-µm radius hollow borosilicate glass sphere coated with 50 nm of Ag (from Cospheric Technologies), which will function as one-half of the Casimir cavity, is glued to one end of the serpentine wire using Ag epoxy (Lake Shore Cryotronics, cured by heating at 80 °C for 3 h). This provides electrical connection between the wire and the surface of the sphere. (3) The wire is picked up by the end opposite to the sphere by gluing it to the end of a glass micro-pipette using UV curable epoxy (v, vi). (4) The wire/sphere is lifted off of the substrate, dipped in UV curable epoxy (Norland Optical NOA81), and positioned over the support spheres. (5) The wire/sphere is lowered using a piezoelectric actuator. Contact is determined by monitoring the noise on the X and Y outputs of the sensor, as with the gluing process shown in Fig. 1b. When contact is made, the epoxy is cured by UV exposure with a 365 nm light-emitting diode source (vii). (6) The other end of the wire which is glued to the micro-pipette is brought over to a pad on the ceramic package and glued down using Ag epoxy. (7) Finally, the wire is released by breaking off the end of the micro-pipette (viii). An example of a fully assembled device can be seen in Fig. 1e. The device used for the results in this paper (along with the external Au-coated plate) can be seen in the optical image in Fig. 2a.

The plate is fabricated from a 300-µm-thick silicon wafer, etched into a tapered shape using photolithography and DRIE, and then coated with 10 nm Cr adhesion layer followed by 150 nm of Au using electron beam evaporation.

### Apparatus and experimental details

The modified ADXL203 is mounted on an XY translation stage attached to an optical breadboard. On the same breadboard is another XYZ stage on which the plate is mounted. This stage has its Z position controlled by a Newport Picomotor stick-slip piezoelectric actuator. An additional Newport NPC3SG piezoelectric stack actuator controls the fine position of the plate in the X direction. The apparatus is contained inside a polystyrene foam container along with a 50 Ω power resistor and resistance temperature detector for Proportional-Integral-Derivative controlled temperature with a 28 °C setpoint. Due to building heaters cycling on and off, the maximum temperature variations inside the enclosure over long periods of time are ∼12 m°C; however, for shorter time periods (2 h or less), the temperature can be held within ∼3 m°C (see Supplementary information S3). The container is set upon an active vibration isolation table (Herzan TS-140).

The potential between the sphere and plate is controlled by grounding the plate and applying a voltage on the sphere through the nano-ribbon wire using a 1 mV resolution power supply (Keithley Instruments). Due to a proprietary polymer coating on the ADXL203 MEMS and the insulating UV curable epoxy, there is no electrical connection between the sphere and the proof-mass.

The sensor voltage output is fed directly into a low-pass filter (Stanford Research Systems SR650) with a 3 Hz cutoff frequency and unity gain. The filter output is then sampled by an ADC (National Instruments NIDAQ).

### Measurement and calibration theory and methods

#### Electrostatic force

As discussed previously, electrostatic forces are present between the sphere and plate metal surfaces, even when the two metals are shorted together. By applying a voltage equal and opposite to the residual potential, this unwanted electrostatic effect can by minimized. Additionally, by applying known electrostatic forces between the plate and the sphere, the force sensitivity of the output can be calibrated.

The forces acting on the sphere are assumed to be only due to electrostatic and Casimir interactions. For the following equations, we define the separation: *d* = *x*_p_ − *x*_s_, where *x*_p_ is the absolute position of the plate and *x*_s_ is the absolute position of the sphere. Assuming a simple electrostatic model, we can write:

1 $$F(d,V_{{\rm{bias}}}) = \frac{{\varepsilon _0\pi R(V_0 + V_{{\rm{bias}}})^2}}{d} + F_{{\rm{Casimir}}}(d),$$

where ε_0_ is the permittivity of free space, *V*_0_ is the residual potential, *V*_bias_ is the applied DC voltage between the sphere and plate, and *R* is the radius of the sphere. Equation 1 uses the PFA for a sphere-plate geometry, which assumes *d* « *R*. At large separations (typically > 200 nm) and large applied voltages (*V*_0_ + *V*_bias_ > 100 mV), the Casimir force term is negligible, and the force scales as *V*_bias_^2^. The residual potential can be measured by sweeping the bias voltage and finding the value of *V*_bias_ at which the force is minimized. At this minimum, the applied bias is equal and opposite to the residual potential.

The sensor outputs an analog voltage, so to get a measurement in units of force, a calibration must be performed. Because of the linear response of the device, the output signal, *S*, is proportional to the force applied on the proof-mass by a constant, *γ*:

2 $$F\left( {d,V_{{\rm{bias}}}} \right) = \gamma {\mathrm{S}}\left( {d,V_{{\rm{bias}}}} \right).$$

For large separations and voltages, we can ignore the Casimir term in Eq. 1 and can now write:

3 $$S\left( {{\mathrm{d,}}V_{{{\rm bias}}}} \right) = \frac{1}{\gamma }\frac{{\varepsilon _0{\mathrm{\pi }}R(V_0 + V_{{\rm{bias}}})^2}}{d}.$$

We can then write *γ* in terms of the second derivative of the signal with respect to *V*_bias_:

4 $${\mathrm{\gamma = }}\frac{{{{2\rm{\pi} \varepsilon }}_0R}}{d}\left( {\frac{{\partial ^2S}}{{\partial V_{{\rm{bias}}}^2}}} \right)^{{\mathrm{ - 1}}}.$$

From these relations, the residual potential and force sensitivity can be measured by fitting the raw signal data, *S*, to the function *S* = *c*_1_*V*_bias_^2^ + *c*_2_*V*_bias_ + *c*_3_. Using the returned fitting parameters we can calculate:

5 $$V_0 = \frac{{c_2}}{{2c_1}},$$

6 $$\gamma = \frac{{{\mathrm{\varepsilon }}_0{\mathrm{\pi }}R}}{{c_1d}},$$

provided we are in a region where the electrostatic force is dominant over the Casimir force.

The procedure for a single measurement is as follows—first the plate is moved by steps of 20 nm towards the sphere until contact is sensed. The plate is then retracted by 1 µm and *V*_bias_ is swept, tracing out a parabola as shown in Fig. 2c, according to Eq. 3. More sweeps are taken as the plate is moved closer by steps of 100 nm. Every data point is the average of 50,000 samples taken in 0.5 s by the ADC with standard deviations between 0.6 and 0.7 mV. The whole electrostatic measurement takes 3.5 min. Over this period of time, thermal drift is negligible (see Supplementary information S3). Fitting a second-order polynomial to these data sets provides measurements of the residual potential, *V*_0_, as well as the sensitivity, *γ*, according to Eqs. 5 and 6. These values are plotted in Fig. 2d.

#### Casimir force

For ideal conditions (absolute zero temperature and perfectly smooth infinitely conducting surfaces), the Casimir force between two plates of area *A* is given by ref. ^6^ as:

7 $$F_{\mathrm{C}}^{{\mathrm{0,PP}}}(d) = \frac{{\hbar c\pi ^2A}}{{240d^4}},$$

where *ħ* is Planck’s reduced constant, *c* is the speed of light in vacuum, and *A* is the overlap area between the surfaces. Using PFA for a sphere-plate geometry, this becomes:

8 $$F_{\mathrm{C}}^0(d) = \frac{{\hbar c\pi ^3R}}{{360d^3}},$$

provided *d* « *R*, as in the electrostatic case. In addition to this ideal case, we consider a corrected model from Geyer et al.^25^, which accounts for more realistic physical effects such as non-zero temperature and the finite conductivity of both metallic surfaces. In this model, a perturbation expansion in powers of the relative penetration depths of electromagnetic oscillations into each metal (using a plasma model) provides a corrected equation for the Casimir force given as:

9 $$F_{\mathrm{C}}^{\mathrm{P}}\left( d \right) =	 \hskip 2pt F_{\mathrm{C}}^0\left( d \right)\left[ 1 + \frac{{45\zeta \left( 3 \right)}}{{{\mathrm{\pi }}^3t^3}} - \frac{1}{{t^4}} - 2\frac{\delta }{d}\left( {2 - \frac{{45\zeta \left( 3 \right)}}{{{\mathrm{\pi }}^3t^3}} + \frac{2}{{t^4}}} \right)\right. \\ 	\left. + \frac{{72}}{5}\frac{{\delta ^2}}{{d^2}} - \frac{{320}}{7}\frac{{\delta ^3}}{{d^3}}\left( {1 - \frac{{2{\mathrm{\pi }}^2}}{{105}}\left( {1 - 3\kappa } \right)} \right)\right. \\ 	\left. + \frac{{400}}{3}\frac{{\delta ^4}}{{d^4}}\left( {1 - \frac{{326{\mathrm{\pi }}^2}}{{3675}}\left( {1 - 3\kappa } \right)} \right)\right].$$

Here, *ζ* is the Reimann zeta function, *t* is a parameter given by *t* = (*ħc*)(2*k*_B_*Td*)^−1^, and *δ* and *κ* are optical parameters given by:

10 $$\delta \equiv \frac{{\delta _{{\rm{Au}}} + \delta _{{\rm{Ag}}}}}{2},$$

11 $${\mathrm{\kappa }} \equiv \frac{{\delta _{{\rm{Au}}} +\delta _{{\rm{Ag}}}}}{{\left( {\delta _{{\rm{Au}}} + \delta _{{\rm{Ag}}}} \right)^2}},$$

where *δ*_Au_ and *δ*_Ag_ are the effective penetration depths of the electromagnetic oscillations into each metal film given by *ħc*/*ω*_p_, in which we have used *ω*_p_ = 9 eV for Au and *ω*_p_ = 8.6 eV for Ag^35^. Equation 9 is a limiting case of this theory in which 1/*t* « 1, which is a valid approximation at our operating temperature (*T* = 301.15 K) and *d* < 1 µm. Finally, a second-order correction for surface roughness is included:^7,36^

12 $$F_{\mathrm{C}}^{\mathrm{R}}\left( d \right) = F_{\mathrm{C}}^{\mathrm{P}}\left( d \right)\left[ {1 + 6\left( {\frac{{A_{\mathrm{r}}}}{d}} \right)^2} \right],$$

where *A*_r_ is the stochastic RMS roughness amplitude of both surfaces. The surface roughness of the plate and sphere surfaces were found to be 2 and 8 nm, respectively, using AFM (supplementary information S1). The total RMS roughness used in this model is *A*_r_ = (*A*_r,sphere_^2^ + *A*_r,plate_^2^)^1/2^ = 8.25 nm.

Immediately following the electrostatic measurement, the plate is retracted back 1 µm away from the sphere and then stepped forward by 1 nm increments as *V*_bias_ is adjusted according to the linear fit, ensuring that the first term of Eq. 1 is minimized at every position. At each plate position, 50,000 samples are taken in 0.5 s using the ADC. This measurement takes 20 min, which requires that the enclosure temperature remain within 3 m°C to avoid unwanted thermal drift (see Supplementary information S3).

After subtracting the zero-force signal (*c*_3_), the measured data is scaled to units of force using the calibration factor *γ*(*d*) measured from the electrostatic data. It is then fit to either the ideal Casimir theory (Eq. 8) or the corrected Casimir theory (Eq. 12), with *x*_s_ as the only free parameter. These results can be seen in Fig. 3.

## Supplementary information

Supplementary information
