# Supplementary material for: Building a Casimir metrology platform with a commercial MEMS sensor
Source: Microsyst Nanoeng. 2019 Apr 22;5:14. doi: 10.1038/s41378-019-0054-5 (PMC6475642; doi:10.1038/s41378-019-0054-5)
Supplement: Supplementary file 1 — Supplementary information [file 41378_2019_54_MOESM1_ESM.doc]

– ***Supplementary Information*** –

**Building a Casimir Metrology Platform with a**

**Commercial MEMS Sensor**

Alexander Stange^1^, Matthias Imboden^6^, Josh Javor^2^, Lawrence K. Barrett^1^,

and David J. Bishop^1,2,3,4,5^

*^1^Division of Material Science and Engineering*

*^2^Department of Mechanical Engineering*

*^3^Department of Physics*

*^4^Department of Electrical and Computer Engineering*

*^5^Department of Biomedical Engineering*

*Boston University,*

*Boston, MA 02215*

*^6^Institute of Microengineering, École Polytechnique Fédérale de Lausanne*

*2000 Neuchâtel, Switzerland*

**Figure S1** shows the typical surface roughness of the materials used in the construction of our device. The gold spheres had abnormally distributed roughness and tended to contain large (> 1 μm) debris and were therefore only used for structural purposes. The silver spheres and gold coated silicon plate showed much lower RMS roughness and were used to construct the Casimir cavity. The data shown are taken of samples prepared in the same manner as those used in the experiment but are not scans of the specific surfaces in our Casimir cavity.

**Figure S1:** SEM and AFM characterization of typical spherical and planar surfaces used in the device.

**Figure S2** shows the effect that the metal plate has on the ADXL203 sensor output due to its interaction with the fringe fields of the sensing electrodes. These scans were taken with the plate starting at approximately 12 μm above the proof-mass (black) and decreasing in height by 1 μm for each dataset. At each height, the plate is scanned by 3 μm in the X direction with the piezo stack actuator. Data was taken over a portion of the proof-mass far away from the sphere with the sphere and plate both grounded.

**Figure S2:** Data illustrating the effect of the plate height on the sensor output due to interactions with the fringe fields of the sensing electrodes.

**Figure S3** displays the stability of the ADXL203 sensor output over a long time period. The periodicity of both datasets is a result from the heaters in the building turning on and off every two hours. Long term drift in the ADXL signal is also apparent. Between these unstable periods (red inset), our PID system is able to hold the temperature to within 3 m°C for 0.5 hours with is the length of a full experiment. For this data, the plate is placed 1 μm away from the sphere and a bias of 1 V is applied between the two surfaces resulting in an applied electrostatic force of approximately 1 nN.

**Figure S3:** Stability of the temperature and the sensor output in the temperature controlled enclosure over a 6.5 hour period.
